# *Alouatta* Trichromatic Color Vision: Cone Spectra and Physiological Responses Studied with Microspectrophotometry and Single Unit Retinal Electrophysiology

**DOI:** 10.1371/journal.pone.0113321

**Published:** 2014-11-18

**Authors:** Luiz Carlos L. Silveira, Cézar A. Saito, Manoel da Silva Filho, Jan Kremers, James K. Bowmaker, Barry B. Lee

**Affiliations:** 1 Instituto de Ciências Biológicas, Universidade Federal do Pará, Belém, Pará, Brazil; 2 Núcleo de Medicina Tropical, Universidade Federal do Pará, Belém, Pará, Brazil; 3 Department of Ophthalmology, University Hospital Erlangen, Erlangen, Germany; 4 Division of Visual Science, Institute of Ophthalmology, University College London, London, England, United Kingdom; 5 State College of Optometry, State University of New York, New York, New York, United States of America; 6 Max Planck Institute for Biophysical Chemistry, Göttingen, Germany; University of Sussex, United Kingdom

## Abstract

The howler monkeys (*Alouatta* sp.) are the only New World primates to exhibit routine trichromacy. Both males and females have three cone photopigments. However, in contrast to Old World monkeys, *Alouatta* has a locus control region upstream of each opsin gene on the X-chromosome and this might influence the retinal organization underlying its color vision. Post-mortem microspectrophotometry (MSP) was performed on the retinae of two male *Alouatta* to obtain rod and cone spectral sensitivities. The MSP data were consistent with only a single opsin being expressed in each cone and electrophysiological data were consistent with this primate expressing full trichromacy. To study the physiological organization of the retina underlying *Alouatta* trichromacy, we recorded from retinal ganglion cells of the same animals used for MSP measurements with a variety of achromatic and chromatic stimulus protocols. We found MC cells and PC cells in the *Alouatta* retina with similar properties to those previously found in the retina of other trichromatic primates. MC cells showed strong phasic responses to luminance changes and little response to chromatic pulses. PC cells showed strong tonic response to chromatic changes and small tonic response to luminance changes. Responses to other stimulus protocols (flicker photometry; changing the relative phase of red and green modulated lights; temporal modulation transfer functions) were also similar to those recorded in other trichromatic primates. MC cells also showed a pronounced frequency double response to chromatic modulation, and with luminance modulation response saturation accompanied by a phase advance between 10–20 Hz, characteristic of a contrast gain mechanism. This indicates a very similar retinal organization to Old-World monkeys. Cone-specific opsin expression in the presence of a locus control region for each opsin may call into question the hypothesis that this region exclusively controls opsin expression.

## Introduction

In catarrhine primates (Old-World monkeys, apes, and humans), the genes for the middle- (M) and long-wavelength (L) sensitive opsins form a tandem array on the X chromosome and, together with the short-wavelength (S) cone opsin, coded on chromosome 7, form the basis for trichromacy [1]–[3]. In diurnal platyrrhine primates (New-World monkeys), usually only one longer wavelength opsin gene is found on the X chromosome but, depending of the species, two or more alleles are present [4], [5]. This means that all males are dichromats but if a female possesses two different alleles on her X chromosome pair, trichromatic color vision can be attained. This has been demonstrated using different behavioral methods [6], [7] and physiologically recordings of opponent cone responses from retinal ganglion cells and lateral geniculate nucleus (LGN) relay neurons [8]–[11].

A major exception is the *Alouatta*. Electroretinographic studies in this platyrrhine species showed that in males and females both M- and L-opsins are present [12], and there is evidence in males and females for behavioral trichromacy [13]. The *Alouatta* L- and M-photopigments have spectral absorption maxima at about 560 and 530 nm, close to those of catarrhines [12], but there may be some inter-individual variability [14]. The genes coding these opsins are present in a tandem array on the X chromosome, as in catarrhines, but with a different structure [4], [14], [15].

In catarrhines, a single locus control region (LCR) is situated upstream of the tandem gene array, and this is thought to control which gene is expressed [4], [15]. In the *Alouatta*, a LCR is present upstream of each opsin gene, suggesting that the opsin gene and upstream mechanisms are both duplicated [4], [15]. As a result, *Alouatta* trichromacy may have a different etiology compared to catarrhines [4], [15].

Anatomically, the retinae of diurnal platyrrhines are very similar to those of catarrhines [16]–[19]. Physiologically, early studies performed, for example, on the squirrel monkey, demonstrated the differences in color opponency in LGN between males and females [20]. Later, physiology performed in further platyrrhine species (*Cebus* sp., *Saimiri* sp., and *Callithrix* sp.) confirmed color opponency in the retinal and LGN cells of trichromatic females, and suggested a pattern that is broadly similar to that of catarrhines, with low achromatic and high chromatic contrast sensitivity of PC cells, and higher contrast sensitivity of MC cells; dichromatic male platyrrhines also showed a low contrast sensitivity of PC cells, indicating an adherence to a primate pattern [8], [9], [11], [21]–[26].

The retinal anatomy of the *Alouatta* shows primate characteristics in addition to some unique features. Franco and colleagues estimated the cone density for both retinas of one individual and found that they had an extremely high cone density of about 429,000 cones/mm^2^ and 357,000 cones/mm^2^, in the foveola [17]. This density is higher than in any other primate described so far, including humans, for which the highest reported individual value was 324,000 cones/mm^2^ [27]. On the other hand, the retinal ganglion cell distribution in the *Alouatta* is similar to that observed in other diurnal platyrrhines and catarrhines [28].

However, physiological characteristics of the *Alouatta* retina remain unknown. If the LCR controls which opsin is expressed, the dual LCRs in the *Alouatta* could provide a potential for mixed expression of two opsins in the same cone; this seems unlikely in face of the behavioral data, but the mechanisms for expression control in *Alouatta* is unknown. Physiologically, the characteristics of ganglion cells of the male *Alouatta* might be expected to resemble those of catarrhines. We here address these issues. *Alouatta* specimens are rarely available for physiological experiments. This brief note contains limited data from two male animals suggesting that pigment expression is indeed cone specific and that ganglion cells closely resemble the catarrhine pattern. Preliminary reports of these experiments have appeared elsewhere [29], [30].

## Methods

### Ethical statement

All animal experiments were carried out in accordance with the National Institute of Health Guide for the Care and Use of Laboratory Animals (NIH Publications No. 80-23, revised 1996), and were approved by the Ethical Research Committee for Animal Experiments of the Institute of Biological Sciences, Federal University of Pará (#MED004/2008).

### Animals

Two male *Alouatta caraya*, were obtained from the breeding colony of the Centro Nacional de Primatas – CENP (Ananindeua, Pará, Brazil) (Appendix S1). The animals were bred and kept in the CENP facilities until the day the experiment started. There, they were kept in housing conditions permitting social interaction with their conspecifics and submitted to an appropriate feeding regimen and drinking water at libitum. Animal housing conditions, feeding regimen, and health were supervised by the CENP veterinarian staff. The animals were kept in environmental enriched cages as recommended by CENP primatologists.

Each animal room measured 2.50 m x 4.00 m x 2.25 m, 10 m^2^ of total area. Each room pair housed 1 male and 5–6 female *Alouatta*. Houses and rooms were cleaned daily. They were washed with jets of water containing 10% hypochlorite. Animals were moved from one of the paired rooms to the other during cleaning. Clean water was provided for primates in 500 ml animal drinking bottles, two bottles per room. Bottles were refilled 3 times per day. Primate diet consisted of the following items: a) food pellets provided in plates, ad libitum; b) fruits, vegetables, once a day; cooked eggs, twice a week; diluted milk, twice a week. In addition, *Alouatta* were provided, daily with Embauba leaves which are part of the natural diet of these Amazonian primates. All animals housed in the CENP received continuous veterinary care which followed the following protocol: inspection in the first hour every day to observe each animal and to inspect the feeding plates and drinking bottles; thereafter regular inspection until the evening. There are also facilities for primate anesthesia and surgery. The facilities allow primate full veterinary assistance, including X-ray, ultrasound, and complete blood, urine, and stool clinical laboratory examination and testing. Each room is enriched with trunks, branches, and ropes to facilitate primate exercising and escape during disputes and force display. Platforms are available for resting and access for feeding.

On the day of experiment, the animal were sedated with an intramuscular injection of ketamine (*ca* 20 mg kg^−1^) and then transferred to the Biological Sciences Institute of the Federal University of Para.

### Microspectrophotometry

Two eyes, one eye of each animal, were lightly fixed in 2% glutaraldehyde and subsequently examined. All procedures were carried out under dim red light. The eyes were enucleated, hemisected, and the anterior portion removed. One or two small pieces of retina, about 1 mm square, were taken from the fovea and parafoveal regions each, placed on a coverslip and mounted in saline containing 10% dextran, before being squashed with a second coverslip, which was sealed with wax. Although the tissue was in a relatively poor state of preservation, a small number of cone and rod outer segments were identified.

Microspectrophotometric recordings from these cells were performed in the conventional manner using a Liebman dual-beam microspectrophotometer [2], [6], [31]. Spectra were recorded at 2 nm intervals from 750 to 350 nm and from 351 to 749 nm on the return scan. The outward and return scans were averaged. A baseline spectrum was measured for each cell, with both beams in an unoccupied area close to the cell, and this was subtracted from the intracellular scan to derive the final spectrum. Two baseline scans were recorded for each cell and averaged. All cells were fully bleached with white light and post bleach spectra recorded. Records that either had very low absorbance or were clearly distorted were discarded. The wavelength of maximum absorbance (λ_max_) of both the absorbance spectra and difference spectra were determined by a standard computer program that best fits a visual pigment template to the right hand limb of the spectra [2], [6].

A standardized computer program was used to estimate the wavelength of maximum absorbance (λ_max_) as fully detailed in Mollon and colleagues [6]. In summary, each of 20 relative absorbance values on the long-wave limb of the absorbance spectrum (corresponding to a 40 nm segment of the record and to absorbance in the range approximately 45–90% of the maximum for the cell) was then referred to a standard template curve to obtain an estimate of the λ_max_: this operation amounts to finding the spectral location of the standard curve that gives the percentage absorbance value under consideration. The 20 individual estimates were then averaged to give the values given in the paper. The segment of the right hand limb of the spectrum was used because it includes the steepest part of the photopigment’s absorbance spectrum and so small changes in the wavelength correspond to large changes in absorbance, and secondly, the short-wave region in microspectrophotometric measurements is the most variable owing to factors including wavelength dependent scattering and the potential presence of photoproducts.

Because the absorbance spectra of visual pigments become narrower towards shorter wavelengths, an empirical template has to be used to estimate the λmax. The template used in this study, and in all previous microspectrophotometric measurements from this lab recorded from both Old and New World monkeys, was based on the classical Dartnall nomogram expressed in units of λ^¼^, an empirical relationship that can be used over the limited spectral range of primate rods and L and M cones. More recent empirical templates, such as those by Govardovskii and colleagues [32]), that can be used over the full spectral range, show, over the limited spectral range used here, little if any variation from the template employed in this study.

### Electrophysiology

Animals were initially anaesthetized with an intramuscular injection of ketamine (*ca* 20 mg kg^−1^). Later, anesthesia was maintained by intravenous infusion of sufentanyl (0.5–4 µg kg^−1^ h^−1^). EEG and ECG were continuously monitored to ensure adequate depth of anesthesia and analgesia. Muscular paralysis was achieved by infusion of 5 mg kg^−1^ h^−1^ of gallamine triethiodide, and the animals were respired with O_2_ to which *ca* 1–2% CO_2_ had been added. End-tidal *P*_CO2_ was kept between 4 and 5% and body temperature maintained within the normal limits. The eye was prepared in a similar way to the eye of the macaque and *Cebus*, and recording of ganglion cell activity was performed as in those species [9], [33]. Briefly, the conjunctiva was sewn to a ring for stabilization and a cannula inserted behind the limbus. A tungsten-in-glass microelectrode was passed through the cannnula, and on touching the retinal surface the electrode was permitted to penetrate into the ganglion cell layer and the activity of a single neuron isolated. A two-dimensional gimbal system, rotating about the point of entry to the globe, permitted us to target different retinal regions. Following completion of recordings, animals were killed with an overdose of barbiturate, Thionembutal (Abbott, Abbott Park, Illinois, USA), 35 mg/kg or higher. Death was assessed by cessation of ECG activity.

Visual stimuli were presented using a Maxwellian view system with red and green diodes (LEDs) as light sources [8], [34]. The system could be rotated about the pupil in order to center the stimulus on a cell’s receptive field. The temporal waveforms for the LEDs were generated by a computer through 12-bit digital-to-analog converters. The LEDs were driven by a frequency-modulated pulse train which provided a highly linear relationship between driving voltage and light output. The emission spectra of the LEDs were measured with a spectroradiometer (Model pro-703/PC, Photo Research, Burbank, CA, USA). The dominant wavelengths of the LEDs were 638 and 554 nm. The mean luminances of LEDs were set using heterochromatic flicker photometry (HFP) matches by an observer whose spectral luminosity function matched the 2 deg Judd’s spectral sensitivity [35]. Mean chromaticity was 595 nm, retinal illuminance levels was *ca* 2000 Td, but because of the small *Alouatta* eye the retinal flux per Troland is about 2 times that in humans.

For each cell, we recorded responses to four different stimulus series to extend cell classification and investigate the retinal mechanisms for *Alouatta* trichromacy. 1) Responses to 400 ms luminance and chromatic pulses were measured to assess the time course of responses; luminance pulses were incremental or decremental; chromatic pulses were redward or greenward. The cells’ spectral sensitivity and cone opponency were assessed by measuring responses to sinusoids in two different protocols.

2) In a modified HFP protocol, the relative modulation depths of a 638 nm red diode and a 554 nm green diode were varied while keeping mean chromaticity and mean luminance constants [36]. Non-opponent cells show a null or response minimum at a particular 638/554 nm ratio while red-green opponent cells show a vigorous response at or close to that same ratio [9], [33], [36], [37]. Two to four (4.88–39 Hz) temporal frequencies were measured.

3) The cells’ spectral sensitivity and cone opponency were also investigated using a phase protocol [38], [39]. The relative phase of the luminance modulation of the 638 nm and 554 nm diodes was varied, with constant modulation amplitude. A phase of ±180 deg corresponds to chromatic modulation and of 0 deg to luminance modulation. For the phase protocols, MC cells were studied at 20 or 50% modulation contrast while PC cells were studied at 50 or 100% modulation contrast. Six temporal frequencies (1.22–39 Hz) were measured.

4) MC cells and PC cells modulation transfer functions (MTFs) were obtained by recording cell responses to sinusoidal stimuli at twelve temporal frequencies and multiple contrast levels. Luminance modulation, with the red and green LEDs in phase, was employed for all cells. Responses of color opponent cells to chromatic modulation, with the LEDs out of phase, were also measured. For luminance modulation, luminance contrast was calculated as (L_max_ − L_min_)/(L_max_+L_min_). For chromatic contrast, cone contrast was calculated in a similar manner, but using cone absorptions.

For all sinusoidal modulation conditions, about 6 s of activity was averaged for each condition, and first and second harmonic amplitudes and phases were extracted.

## Results

### Microspectrophotometry

**Figure 1:**
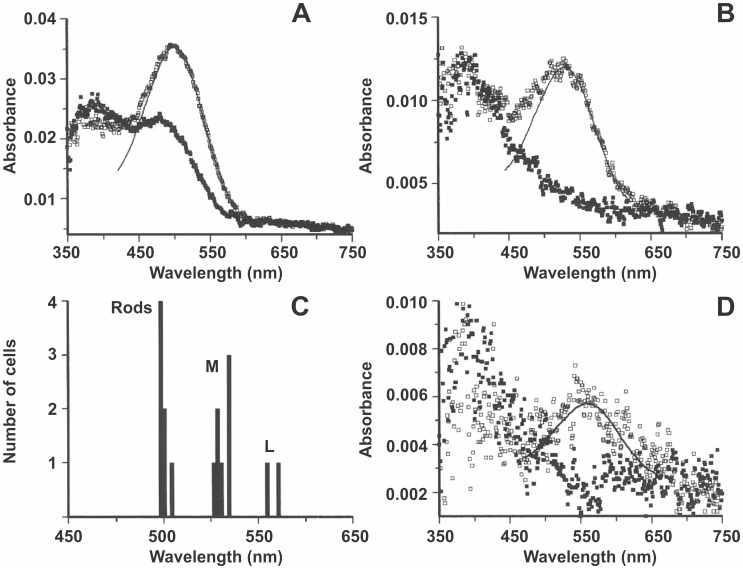
Absorbance spectra of visual pigments from *Alouatta caraya*. Plots in (**A**), (**B**), and (**D**) show, respectively, rod, middle-wave sensitive cone, and long-wave sensitive cone pre-bleach (open symbols) and post-bleach (filled symbols) spectra. Histogram in (**C**) shows spectral distribution of λ_max_ of individual rods and cones obtained from absorbance spectra.

Ten cone outer segments were analyzed and these fell into two clear populations, eight with λ_max_ close to 530 nm (529.5±5.5 nm) and two with λ_max_ around 557 nm (557.5±4.9 nm). All these outer segments were fully bleached after exposure to white light. No S cones were identified. Seven rod outer segments gave a mean λ_max_ of 499.8±1.8 nm, but bleaching with white light revealed, in addition to the typical ‘retinal’ peak at about 370 nm, a stable photoproduct absorbing around 465 nm, presumably metarhodopsin III, which is sometimes seen in lightly fixed tissue. The absorbance of the cone outer segments was low, 0.009 and 0.004 for the MWS and LWS cones, respectively, whereas the rods, which were better preserved, had a transverse absorbance of 0.029. A summary of the microspectrophotometric results is presented in Figure 1. These data are consistent with selective expression of the M and L opsins in different cones.

Although the data are limited, the records fall into two clear spectral groups of L and M cones, but as with all primate microspectrophotometric data, we cannot explicitly exclude the possibility of a limited degree of co-expression within each cone class. No published microspectrophotometric data from monkeys or humans has ever explicitly tested for co-expression. It would require very careful and somewhat difficult partial bleaching experiments due to the small spectral separation in these animals.

### Electrophysiology

We recorded from 34 ganglion cells from the retinae of the two animals (Dataset S1). Cell receptive fields were plotted on a tangent screen and were located within 10 deg of the fovea. Initial cell classification was performed with flashed spots of different colors and gave similar results to those previously obtained from recordings from the retinae of macaques and *Cebus* [9], [33], [37], [40]. Cells that were classified as PC cells at this stage could be clearly identified by their vigorous response, either excitatory or inhibitory, to green and red lights. KC cells with excitatory S-cone input strongly responded to short wavelength light. Cells that were classified as MC cells could be identified by their good response to low achromatic contrast. Using these criteria, we identified 6 PC cells, 1 S-cone On cell, and 17 MC cells. The remaining 10 cells of our sample remained unclassified mostly because these cells were not recorded for the necessary time for clear classification. Further support for these classifications was provided by the quantitative analyses as described below.

**Figure 2:**
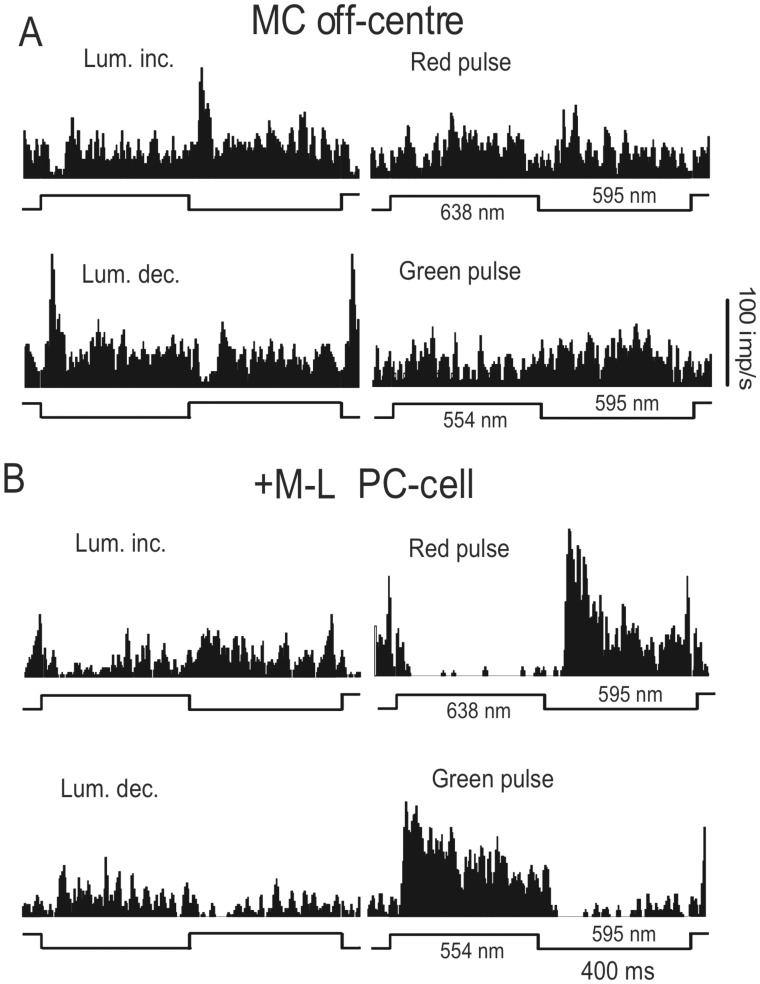
Ganglion cell responses to luminance and color step changes. Responses to 50% contrast, 400 ms step changes in luminance (increment or decrement) and color (redward or greenward pulses) of an MC off-centre cell (A) and a +M-L PC off-centre cell (**B**). The MC cell showed phasic responses to luminance changes and little responses to chromatic pulses. The PC cell showed strong tonic responses to the greenward pulse, was strongly inhibited by the redward pulse, and showed small tonic responses to luminance changes. Histograms are averages of 20 sweeps; 4 ms/bin.

We then measured cell responses to 400 ms step changes in luminance (increment or decrement) and color (redward or greenward pulses) at various contrast levels. Figure 2 shows the responses of an MC off-centre cell (A) and a +M-L PC off-centre cell (B). The MC cell showed strong phasic responses to luminance changes and little response to chromatic pulses. The PC cell showed strong tonic response to the green pulse, was strongly inhibited by the red pulse, and showed small tonic responses to luminance changes. These responses are very similar to those of macaque ganglion cells [33], [36], [37].

**Figure 3:**
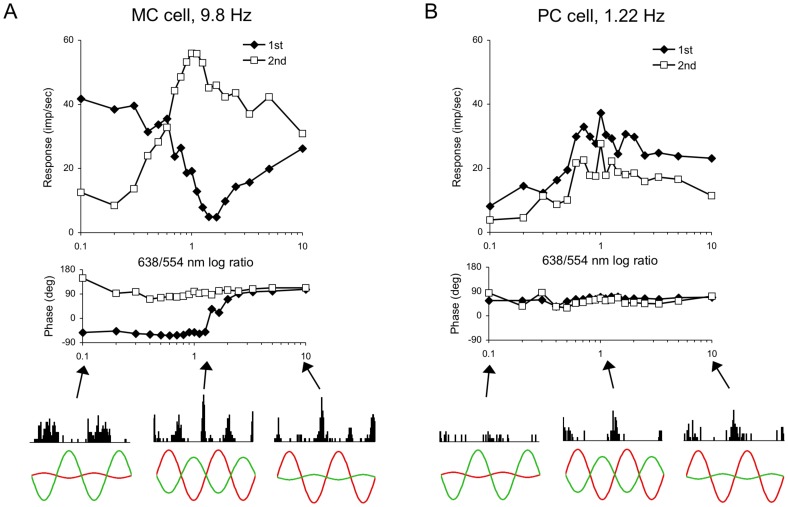
Ganglion cell responses to heterochromatic flicker photometry stimuli. (**A**) MC and (**B**) PC cell responses to heterochromatic stimuli. HFP protocol: The relative modulation depths of counterphase modulated 554 and 638 nm LEDs were manipulated. MC and PC cells were stimulated at 9.8 Hz and 1.22 Hz, respectively. Stimulus size was, 4 deg mean retinal illuminance was 2000 Td. Each data point was obtained from the Fourier analysis on averaged responses in episodes each lasting 6 s. Top and middle panels: response amplitude and phase as a function of the red/green amplitude ratio, respectively. Filled diamonds and empty squares represent the parameters for response first and second harmonics, respectively. Bottom panels: the histograms illustrate actual averaged responses to two modulation cycles, the arrows point at the employed red/green ratios for the histograms, and the red and green curves represent the temporal luminance modulation of the red and green lights for each stimulus condition. In the HFP protocol, *Alouatta* MC cell responses displayed a minimum, coinciding with a sudden shift in phase, when the modulation ratio of the red and green lights was slightly larger than unity. The amplitudes of the second harmonic component peaked around a contrast ratio of unity, where it was substantially larger than the amplitude of the first harmonic component, whilst its phase remained stable. The resultant frequency doubled response can be observed in the corresponding histogram. *Alouatta* PC cells showed vigorous response and no phase shift for nearly all stimulus conditions.

**Figure 4:**
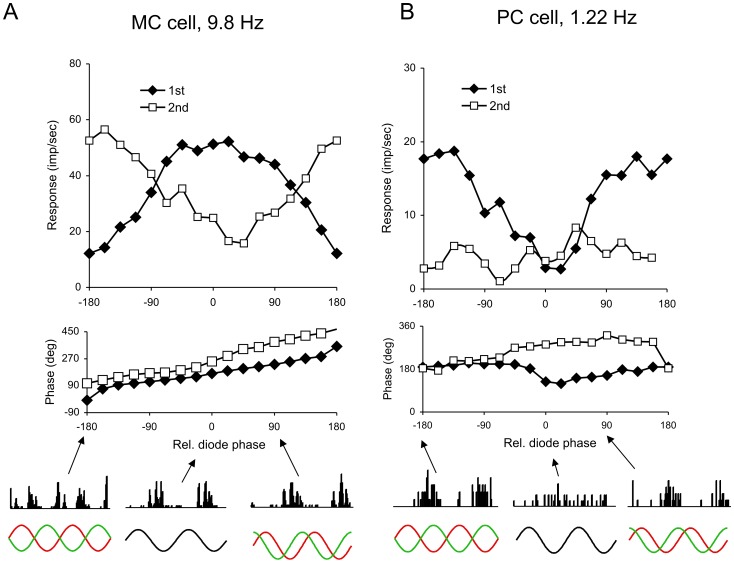
Ganglion cell response to stimulus phase changes. (**A**) MC and (**B**) PC cell responses to heterochromatic stimuli. Phase protocol. The relative phases of the 554 and 638 nm LEDs were modulated with fixed modulation depths. MC and PC cells were stimulated at 9.8 Hz and 1.22 Hz, respectively. Stimulus size was 4 deg, mean retinal illuminance was 2000 Td. Response amplitudes (top panels) and phases (middle panels) are shown as a function of the phase difference between the luminance modulation in the red and green LEDs. Filled diamonds and empty squares represent the parameters for response first and second harmonics, respectively, extracted by Fourier analysis. Bottom panels: the histograms illustrate actual responses to two cycles of modulation, the arrows indicate the red/green phase difference for the histograms, and the red and green curves represent how the phase of the red and green lights changed for each stimulus condition. Luminance modulation corresponds to a relative phase of 0 deg, chromatic modulation to a relative phase of ±180 deg. MC cells responded to the phase protocol with a maximal response first harmonic amplitude when the green and red lights were modulated in phase and their response phase changed continuously with the phase difference between the modulation in the two LEDs. PC cells displayed a minimal response when the green and red lights were modulated in phase and their response phases changed abruptly in the region of minimal response.

Figures 3 and 4 shows *Alouatta* MC and PC cell responses to heterochromatically modulating stimuli. As described in the Methods section, an amplitude protocol based on HFP (Figure 3) and a phase protocol (Figure 4) were used to characterize the spectral sensitivity and color-opponency of ganglion cells.

*Alouatta* MC cells’ responses to the HFP protocol displayed a minimum when the contrast ratio of the counterphase-modulated red to green lights was changed. A ratio of unity would suggest a spectral sensitivity similar to the human photopic luminosity function (V_λ_). As an example, Figure 3A shows responses of an MC cell to 9.8 Hz stimuli; response amplitude and phase are plotted against the relative modulation of the red and green LEDs. There is a minimal response of the MC cell first harmonic (filled diamonds) amplitude (upper panel), coinciding with a sudden shift in phase of about 180 deg (middle panel), at a relative amplitude of the red and green LEDs of about 2. *Alouatta* MC cells also showed a pronounced frequency-doubled response, expressed by the presence of a substantial second-harmonic component with an amplitude that peaked near a contrast ratio of unity (Figure 3A, upper and middle panels, empty squares) whilst phase remained stable. The histograms below the plots illustrate actual responses to two cycles of modulation (Figure 3A, lower panel). Arrows indicate the R/G values for the histograms. The frequency-doubled response is clear in the central histogram.

On average, response minima of MC cells from the two animals tested was close to a luminance ratio between the red and green diodes of one (mean: 1.08, s.d.: 0.26, n = 16) but the two animals differed significantly in mean null values (means 1.25 (n = 9), 0.86 (n = 7), p<0.02, t test). It is established that HFP luminance nulls of humans is related to relative L/M cone numerosity in the retina [41], [42], but the mechanisms controlling relative numerosity are poorly understood; with the different genetic foundation in the *Alouatta* this problem is compounded.

The frequency-doubled response to chromatic modulation of MC cells in the *Alouatta* was very obvious as in the example in Figure 3. As in the macaque [36], it was not present at higher temporal frequencies (39 Hz), with some cell-to-cell variability. This is taken up below.

*Alouatta* PC cells showed vigorous responses to nearly all heterochromatic stimuli; the first harmonic component to 1.22 Hz stimuli (Figure 3B filled diamonds) displayed a broad peak at ratios around unity (upper panel). The phases were similar for all ratios (middle panel). The second harmonic-response follows the first harmonic amplitude but is always smaller and thus is related to response shaping (Figure 3B, upper and middle panels, empty squares). Again, the histograms set below the plots illustrate actual responses to two cycles of modulation (Figure 3B, lower panel).

In conclusion, the responses of *Alouatta* MC cells and PC cells to the HFP protocol were similar to those of their counterparts found in the retinas of macaques and trichromatic *Cebus* [9], [33], [36], [37].

In the phase protocol, rather than changing the relative amplitude of modulated lights, their relative phase is varied. This protocol can determine relative weights and temporal properties of cone mechanisms in ganglion cell responses [39]. At 10 Hz and above, macaque MC cells respond to the phase protocol with a first-harmonic maximum when the green and red lights are in phase; their responses reach a minimum when the green and red stimuli are modulated in counterphase. At lower temporal frequencies, the phase of minimum response moves away from zero as in human psychophysical results [38], [39], [43]. At higher frequencies, a similar pattern was found in *Alouatta* MC cells, as shown in Figure 4A. Response amplitude and phase have been plotted as a function of the relative phase of the two LEDs. There is a response minimum near +/−180 deg. The response phase changes gradually with a more rapid transition near +/−180 deg. The second-harmonic response is seen to be very vigorous at this phase. In the macaque [39], second-harmonic response components were sometimes seen but were seldom large enough to disturb analysis of first-harmonic components. In the sample of *Alouatta* cells, frequency-doubled components were very prominent and made analysis of first-harmonic components noisy, especially at lower temporal frequencies. To validate this observation, we compared the ratio of luminance 1^st^ harmonic to chromatic 2^nd^ harmonic responses for samples of cells from the two species, using very similar stimuli (50% modulation contrast, 4.88 Hz, same LED peak wavelengths and mean luminance; macaque data were taken from previous work [34]. Ratios were 2.65 (s.d. 1.42, n = 13) for macaque and 0.758 (sd. 0.31, n = 13) for *Alouatta*, a highly significant difference (p<0.001, t test). This would suggest a species difference.

PC cells responded to the phase protocol with a minimum when the green and red lights were in phase; their responses increased with increasing phase difference between the green and red stimuli and were maximal when the two modulated in counterphase (Figure 4B, upper panel). In addition, the cell response showed an abrupt change in phase in the region of minimum response (Figure 4B, middle panel). These results suggest responses of *Alouatta* MC and PC cells were similar to those of the corresponding ganglion cells found in the retinas of macaques [39] and trichromatic *Cebus* [9], although differences in detail are likely to be present. There appeared to be more variability in the opponent weighting for PC cells than in similar macaque data, but our cell sample was not large.

**Figure 5:**
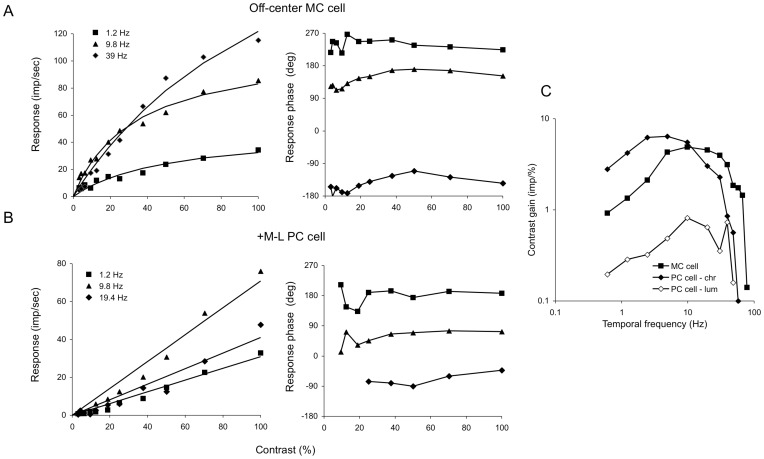
Ganglion cell response to stimulus contrast. (**A–B**) Response amplitude (left panels) and phase (right panels) as a function of stimulus luminance contrast for *Alouatta* MC cell (**A**) and PC cell (**B**). The results obtained with three temporal frequencies are given: 1.2 Hz (filled squares), 9.8 Hz (filled triangles), and 39 Hz (filled diamonds) for the MC cell; 1.2 Hz (filled squares), 9.8 Hz (filled triangles), and 19.4 Hz (filled diamonds) for the PC cell. Data representing response amplitude as a function of contrast have been fitted with Naka-Rushton functions. MC cell vigorously responded to low levels of luminance contrast, but responses rapidly saturated accompanied by advancement in response phase, especially at intermediate and high temporal frequencies. PC cells were quite insensitive to low levels of luminance contrast, but the responses did not exhibit saturation or phase advancement. (**C**) Temporal modulation transfer functions (temporal MTFs) for *Alouatta* MC cells (average of 7 cells) stimulated with luminance sinusoids (filled squares) and a PC cell stimulated with luminance (empty diamonds) and averaged responses of 4 cells to red-green (filled diamonds) sinusoids. Contrast gain was defined as the initial slope of the Naka-Rushton functions fitted to the amplitude versus contrast data such as those illustrated in the left panels (**A–B**). Michelson contrast and cone contrast were used for the luminance and red-green chromatic temporal MTFs, respectively. MC cells were much more sensitive than PC cells to temporal luminance modulation at all the temporal frequencies range and vigorously responded to very high temporal frequencies. On the other hand, PC cells were very sensitive to red-green contrast, especially at low and intermediate temporal frequencies.

To explore further the comparison between *Alouatta* ganglion cell responses on the one hand and those of ganglion cells in macaques and in trichromatic female platyrrhines on the other, we employed sinusoidal luminance and chromatic modulation at different temporal frequencies and contrasts. Figure 5A–B illustrates response amplitudes and phases of *Alouatta* MC (Figure 5A) and PC cells (Figure 5B) as a function of contrast; luminance modulation was used for the MC cell and chromatic modulation for the PC cell. Data representing response amplitude as a function of contrast were fitted with Naka-Rushton functions [44]. MC cell vigorously responded to low levels of luminance contrast at frequencies of 5–40 Hz, but responses rapidly saturated accompanied by response phase advancement, especially at intermediate temporal frequencies (9.8 Hz), as seen in the phase plot. PC cells were most sensitive to chromatic contrast (Figure 5B), but responses did not exhibit saturation or phase advance when contrast was increased. Figure 5C illustrates the averaged temporal MTFs that were obtained from *Alouatta* MC cells (n = 7) and PC cells (n = 4). For both cell classes, we obtained luminance temporal MTFs and for PC cells we also obtained red-green temporal MTFs. The MTFs show the contrast gain as a function of temporal frequency, contrast gain being the initial slope (at zero contrast) of the Naka-Rushton functions fitted to the amplitude-versus-contrast data, cone contrast being used for the red-green temporal MTFs. MC cells were much more sensitive than PC cells to temporal luminance modulation across the temporal frequency range studied and responded vigorously to high temporal frequencies. On the other hand, PC cells were most sensitive to red-green contrast, especially at low and intermediate temporal frequencies.

## Discussion

### Physiological substrate for *Alouatta* trichromacy

This work provides direct physiological data on the functional organization of the *Alouatta* retina for comparison with data in other species. The comparison may shed light on basic principles of primate retinal organization and on possible specific adaptations to trichromatic vision in different primate species.

We have found microspectrophotometric signatures of two populations of middle-to-long wavelength sensitive cones bearing separate expressions of photopigments with absorption peaks close to 530 nm and 558 nm absorption peaks. The presence of these two cone types is the receptoral prerequisite for a red-green color opponent pathway in the primate retina [1], [2], [5]. Cone-specific expression of M and L opsins is not unexpected from ERG and behavioral data [12], but it remains unclear how this occurs when each opsin gene has its own LCR.

In addition, we have found post-receptoral red-green color opponent mechanisms with very similar properties of those previously found in macaques [33], [37], trichromatic female *Callithrix* [8] and trichromatic female *Cebus* [9]. Although the cell sample was limited due to the small number of animals available, the general features found in the macaque were apparent.

### Physiological properties of *Alouatta* retinal ganglion cells

*Alouatta* MC and PC ganglion cells exhibit similarities with MC and PC ganglion cells of catarrhine primates. To list these similarities: MC cells were sensitive to luminance contrast, exhibited phasic responses to luminance steps and responded to the phase protocol and HFP protocols as in the macaque. PC cells were less sensitive to luminance contrast, exhibited tonic responses to chromatic pulses, responded to the phase protocol with a minimum response when the green and red lights were in phase and responded to the HFP protocol with an amplitude peak near equal luminance. In addition, two specific features found in macaque ganglion cells were also encountered. MC cells responded to equal-luminance red-green modulation with twice the stimulation frequency [36]; this effect was marked in *Alouatta* MC cells. Also, MC cells showed response saturation and phase advancement with increasing contrast whereas PC cells did not. These latter features of MC cells are characteristic of a contrast gain control mechanisms [45], [46], and found in MC cells but not in PC cells of catarrhines [47], [48] and platyrrhines [8], [9].

Many properties are shared by the MC and PC cells of *Alouatta*, macaques [33], [37], and trichromatic female *Callithrix* [8] and *Cebus* [9]. The MC and PC cells of dichromatic male or female *Callithrix* [8] and *Cebus* [9] also exhibit the similar responses to luminance-modulated stimuli as in their trichromatic conspecifics. In conclusion, although a relatively small number of cells were measured, they displayed response properties that closely match those of other trichromatic species and individuals strongly suggesting that the processing in the retina of *Alouatta* is very similar.

*Alouatta* PC cells comprised the same subclasses found in other trichromats: some were excited by red light and inhibited by green light, while others were excited by green light and inhibited by red light. These responses reflected the excitatory or inhibitory inputs they received from M cones (530 nm absorption peak) or L cones (558 nm absorption peak). No study has yet morphologically identified *Alouatta* bipolar cells, but we suggest from the responses obtained from PC cells that single-cone midget bipolar cells similar to those that have been found in catarrhines [49] and platyrrhines [50], [51] will be present in the *Alouatta* retina.

As complete trichromacy seems to have evolved relatively recently in *Alouatta* (with a different origin compared to catarrhines [4], [15]), it is likely that the receptoral mechanisms were able to take over pre-existent post-receptoral retinal wiring, already utilized in females of the species, and to use them to send trichromatic signals to higher visual centers.

We therefore conclude that the functional organization of the retina is very similar in all anthropoid primates and was probably present before the split between catarrhines and platyrrhines 40 million years ago (MYA). There may well be differences in detail, so that the frequency-doubled MC response to chromatic modulation was very obvious in *Alouatta*, compared to earlier recordings in trichromatic *Callithrix* and *Cebus*. Another feature of trichromatic color vision is cone specificity in the surrounds of PC-cells of macaques [52], [53]. This is present to some degree in the *Callithrix* [54] but the situation in *Alouatta* is unknown. We stress that quantitative differences between retinae of different primate species are likely.

### Contrast sensitivity of *Alouatta* retinal ganglion cells

Temporal MTFs (contrast gain as a function of temporal frequency) obtained from *Alouatta* MC and PC cells followed the same general pattern of temporal MTFs obtained from macaques [34] and *Cebus* [9]. The low luminance contrast gain of PC cells observed in trichromatic primates have been considered to be largely due to mutual cancellation of the opponent cone signals [33], [34], [37]. PC cells of dichromatic and trichromatic *Cebus* monkeys showed similar low gain [9]. PC cells of dichromatic platyrrhines might be described as color blind versions of cone-opponent PC cells of trichromatic primates. The functional significance played by such a numerous, luminance contrast insensitive cell class remains uncertain. In any event, PC cells in male *Alouatta* appear to have properties as in the macaque.

### Evolution of primate trichromacy

Among the possible scenarios for the evolution of trichromatic vision in primates [9], [55], a common view is that polymorphic color vision arose in prosimians and passed to anthropoids before the split between catarrhines and platyrrhines. Full trichromacy then arose independently at least twice, in catarrhines and in the *Alouatta* [4], [56], [57]. The absorption peaks of the two LWS cone opsins found in the two groups are usually similar (Jacobs et al. [12] and the present study), possibly indicating an optimization for red-green color opponency in regular trichromats. Another possibility, originally proposed by Mollon [55], and supported by others based on molecular data [4], [14], [15], is that trichromatic vision in platyrrhines and catarrhines evolved separately. However, the close similarities in retinal anatomy and physiology between the two groups, further underlined here, make, in our view, parallel evolution unlikely.

There are several hypotheses about the evolutionary pressures that might have driven primates to trichromacy and they are generally related to foraging behavior. Stephen Polyak was probably the first to explicitly point out that detecting orange and red fruits against the green foliage would benefit from trichromacy [58]. There have been several studies measuring fruits and leaves surface reflectance and quantifying primate foraging behavior in natural conditions which were then used to provide quantitative data for theories of trichromacy evolution [59]–[64]. These ideas focused in the evolutionary advantage of acquiring a better distinction of reddish fruits against green foliage [65], [66], yellowish dappled with red young nutritive leaves against mature green leaves [62], and reddish falling leaves against healthy green leaves [67]. These possibilities are not mutually exclusive. It should be observed that both catarrhines and *Alouatta* distinguish themselves from the majority of platyrrhines by having a diet mainly based on fruits and on leaves [67], [68]. For the *Alouatta*, there have been reports from field studies of their foraging behavior associated with quantitative analysis in the color domain of surface reflectance underlying the importance of selecting both the appropriate fruits [60], [61] and leaves [64] for their diet.

## Supporting Information

Appendix S1 ***Alouatta*** **housing conditions including feeding regimens and environmental enrichment.**
(DOC)

Dataset S1 **Ganglion cell responses to different stimulus protocol.**
(RAR)

Checklist S1 **The ARRIVE Guidelines Checklist.**
(DOC)
