# Supplementary material for: Alouatta Trichromatic Color Vision: Cone Spectra and Physiological Responses Studied with Microspectrophotometry and Single Unit Retinal Electrophysiology
Source: PLoS One. 2014 Nov 18;9(11):e113321. doi: 10.1371/journal.pone.0113321 (PMC4236167; doi:10.1371/journal.pone.0113321)
Supplement: Appendix S1 — Alouatta housing conditions including feeding regimens and environmental enrichment. (DOC) [file pone.0113321.s001.doc]

**Alouatta housing conditions including feeding regimens and environmental enrichment**

1. **Buildings where animals were kept.** Buildings were designed for keeping and breeding neotropical primates. Each one measured 640 m^2^ and comprised 12 pairs of rooms for primate housing (total of 24 rooms).
2. **Housing for the animals.** Each animal room measured 2.50 m x 4.00 m x 2.25 m, 10 m² of total area.
3. **Number of animals per room.** Each room pair housed 1 male and 5-6 female *Alouatta*.
4. **Cleaning routine.** Houses and rooms were cleaned daily. They were washed with jets of water containing 10% hypochlorite. Animals were moved from one of the paired rooms to the other during cleaning.
5. **Water availability.** Clean water was provided for primates in 500 ml animal drinking bottles, two bottles per room. Bottles were refilled 3 times per day.
6. **Feeding regime.** Primate diet consisted of the following items: a) food pellets provided in plates, ad libitum; b) fruits, vegetables, once a day; cooked eggs, twice a week; diluted milk, twice a week. In addition, *Alouatta were provided*, daily with Embauba leaves which are part of the natural diet of these Amazonian primates.
7. **Veterinary care.** All animals housed in the National Primate Center received continuous veterinary care which followed the following protocol. Inspection in the first hour every day to observe each animal and to inspect the feeding plates and drinking bottles. Thereafter regular inspection until the evening. There are also facilities for primate anesthesia and surgery. The facilities allow primate full veterinary assistance, including X-ray, ultrasound, and complete blood, urine, and stool clinical laboratory examination and testing.
8. **Environmental enrichment.** Each room is enriched with trunks, branches, and ropes to facilitate primate exercising and escape during disputes and force display. Platforms are available for resting and access for feeding.
